# Automated treatment planning as a dose escalation strategy for stereotactic radiation therapy in pancreatic cancer

**DOI:** 10.1002/acm2.13025

**Published:** 2020-10-16

**Authors:** Savino Cilla, Anna Ianiro, Carmela Romano, Francesco Deodato, Gabriella Macchia, Pietro Viola, Milly Buwenge, Silvia Cammelli, Antonio Pierro, Vincenzo Valentini, Alessio G. Morganti

**Affiliations:** ^1^ Medical Physics Unit Gemelli Molise Hospital ‐ Università Cattolica del Sacro Cuore Campobasso Italy; ^2^ Radiation Oncology Unit Gemelli Molise Hospital ‐ Università Cattolica del Sacro Cuore Campobasso Italy; ^3^ Radiation Oncology Department DIMES Università di Bologna ‐ Ospedale S.Orsola Malpighi Bologna Italy; ^4^ Radiology Department Gemelli Molise Hospital ‐ Università Cattolica del Sacro Cuore Campobasso Italy; ^5^ Radiation Oncology Department Fondazione Policlinico Universitario A. Gemelli ‐ Università Cattolica del Sacro Cuore ‐ Roma Italy

**Keywords:** autoplanning, SBRT, SIB, pancreas

## Abstract

**Purpose:**

To assess the feasibility of automated stereotactic volumetric modulated arc therapy (SBRT‐VMAT) planning using a simultaneous integrated boost (SIB) approach as a dose escalation strategy for SBRT in pancreatic cancer.

**Methods:**

Twelve patients with pancreatic cancer were retrospectively replanned. Dose prescription was 30 Gy to the planning target volume (PTV) and was escalated up to 50 Gy to the boost target volume (BTV) using a SIB technique in 5 fractions. All plans were generated by Pinnacle^3^ Autoplanning using 6MV dual‐arc VMAT technique for flattened (FF) and flattening filter‐free beams (FFF). An overlap volume (OLV) between the PRV duodenum and the PTV was defined to correlate with the ability to boost the BTV. Dosimetric metrics for BTV and PTV coverage, maximal doses for serial OARs, integral dose, conformation numbers, and dose contrast indexes were used to analyze the dosimetric results. Dose accuracy was validated using the PTW Octavius‐4D phantom together with the 1500 2D‐array. Differences between FF and FFF plans were quantified using the Wilcoxon matched‐pair signed rank.

**Results:**

Full prescription doses to the 95% of PTV and BTV can be delivered to patients with no OLV. BTV mean dose was >90% of the prescribed doses for all patients at all dose levels. Compared to FF plans, FFF plans showed significant reduced integral doses, larger number of MUs, and reduced beam‐on‐times up to 51% for the highest dose level. Despite plan complexity, pre‐treatment verification reported a gamma pass‐rate greater than the acceptance threshold of 95% for all FF and FFF plans for 3%‐2 mm criteria.

**Conclusions:**

The SIB‐SBRT strategy with Autoplanning was dosimetrically feasible. Ablative doses up to 50 Gy in 5 fractions can be delivered to the BTV for almost all patients respecting all the normal tissue constraints. A prospective clinical trial based on SBRT strategy using SIB‐VMAT technique with FFF beams seems to be justified.

## 1 INTRODUCTION

Pancreatic carcinoma is the fourth leading cause of cancer death in developed countries.^1^ Despite the recent advancements in surgical, chemotherapy, and radiation therapy, the overall survival rates at 1 and 5 years are at 26% and 6%, respectively.^1^ At the time of initial diagnosis, the tumor is usually locally advanced and infiltrates the main blood vessels, such as the superior mesenteric artery, the portal confluence, and the celiac trunk; thus, significantly increasing the likelihood of margin positive resection.

Radiation therapy as local treatment has been utilized as neoadjuvant, adjuvant, or definitive treatment with or without systemic therapy. When the treatment has a neoadjuvant purpose, the aim is to downstage the disease to radical resection, even if initially inoperable and improve local control.^2^, ^3^

However, the presence of radiosensitive surrounding organs at risk (OARs), in particular the duodenum, has limited the delivering of high doses to the target, giving a probability of success of about 25‐30%.^4^ The introduction of intensity‐modulated radiotherapy (IMRT) and, later of, volumetric modulated arc therapy (VMAT) has greatly improved the ability to spare adjacent OARs while delivering a therapeutic dose to the target, with the potential to reduce treatment toxicity and improve local control.^5^, ^6^ In particular, since VMAT demonstrated to maintain similar or improved plan quality with respect to fixed‐field IMRT but with a significant reduction in treatment delivery time, it was recently proposed as an optimal technique for pancreatic cancer treatment.^7^, ^8^, ^9^ Furthermore, modulated techniques allowed the simultaneous delivery of different doses to different target volumes within a single fraction, an approach called simultaneous integrated boost (SIB). This last strategy was found to be more efficient in terms of treatment shortening and radiobiological improved effect as the biological equivalent dose to the tumor increases with higher dose per fraction. For pancreatic cancer, this strategy could deliver a boost dose to the portion of tumor infiltrating the peripancreatic vessels, with the aim of achieving tumor resectability, and a lower dose to the rest of the target volume, avoiding an overdosage to the portion of tumor overlapping the duodenal wall.^10^ The dosimetric feasibility of IMRT and VMAT with SIB for pancreatic cancer has been recently successfully demonstrated for conventional dose fractionation showing excellent tumor coverage, conformity of dose distribution, and sparing of OARs.^11^, ^12^, ^13^

At the same time, the technological advancements in immobilization and imaging, together with the ability to deliver high conformal doses and to account for organ motion have led to a widespread implementation of stereotactic body radiotherapy (SBRT) in a number of clinical settings.^14^ SBRT has garnered a major interest for pancreatic cancer patients since the delivery of ablative doses in a few fractions may improve downstaging and local control and also the shorter treatment time results in an easy integration with chemotherapy. Recent reviews of the literature,^15^, ^16^, ^17^ focused on the use of SBRT for pancreatic cancer in unresectable cases, reported that (i) the survival outcomes of patients treated with radiosurgical doses are similar to those recorded on series based on prolonged chemoradiation, (ii) radiation dose escalation can help prevent local tumor progression, (iii) SBRT can be easily integrated into a regimen of aggressive chemotherapy preventing unnecessary delays, and (iv) SBRT has a great potential for conversion to resectability in patients enrolled on a non‐curative treatment regimen.

The simultaneous application of SBRT, VMAT and SIB techniques and strategies to pancreatic tumors suggests a new possible clinical paradigm, in which high ablative focused doses are delivered in few fractions to the portion of the tumor near the vascular infiltration and lower doses are simultaneously administrated to the rest of the target volume. This aim is very challenging and obtaining quality plans is a demanding task for medical physicists and dosimetrists.

Recently, various algorithms have been proposed for an automatic optimization of the planning procedure and the search for the optimal patient plan. In particular, fully automated VMAT quality plans for head‐neck,^18^, ^19^ prostate,^20^ and for SBRT treatments of liver^21^ and lung^22^ metastasis have been successfully generated for clinical application using the Autoplanning template‐based optimization engine implemented in Pinnacle^3^ treatment planning system (TPS, Philips Healthcare, Fitchburg, WI).

In this study we assessed the feasibility of automated SBRT‐VMAT planning using a SIB approach as a dose escalation strategy for stereotactic radiation therapy in pancreatic cancer. Aiming to maximize the dose delivery to the target vascular infiltration while minimizing the probability for duodenum toxicity, we retrospectively re‐planned 12 patients evaluating the performance of the Autoplanning module for flattened (FF) and unflattened (FFF) photon beams.

## 2 MATERIAL AND METHODS

A total of 12 patients with unresectable pancreatic head carcinoma due to vascular infiltration were included in this retrospective planning study.

### 2.1 Simulation and volumes definition

Patients were simulated supine with arms up using a Vac‐Lok and the Elekta (Elekta (TM), Crawley, UK) stereotactic body frame (SBF) for immobilization. An abdominal compressor attached to the SBF by a rigid arc was used with the aim to minimize the mobility of targets close to the diaphragm by mechanically pressing the patient’s epigastrium. A study on organ motion due to residual respiratory movements was performed during which the extent of tumor displacement caused by respiration was assessed.

For small bowel visualization, 2 cc of oral Gastrografin diluted in ½ liter of water were given to each patient, 30 minutes before CT scans acquisition, for small bowel visualization purposes.

Target volumes and OARs contouring were performed by a radiation oncologist and a radiologist, using the CT simulation performed in the arterial phase. The site of vascular infiltration was identified and the involved vessel was contoured, with a circumferential margin of 5 mm, for the whole craniocaudal extension of infiltration or contact between the gross tumor volume (GTV) and vessel (or vessels, in case of the involvement of more than one vascular structure). This volume was defined as CTVvasc. The CTVvasc plus an anisotropic margin of 5 mm in the craniocaudal direction and 3 mm in the other directions was defined as BTV (boost target volume).

The tumor PTV (PTV) was defined as the GTV plus an anisotropic margin (5 mm in the craniocaudal direction and 3 mm in the other directions) and including the BTV.

The OARs were delineated as indicated in the RTOG atlas.^23^ The duodenum was delineated from the pylorus to the duodeno‐jejunal junction. Then, a PRV_duodenum was defined by adding 5 mm in craniocaudal direction and 3 mm in the other directions. The kidneys, liver, stomach, and spinal cord were also outlined from 20 cm above the GTV cranial margin to 20 cm below the GTV caudal margin.

To quantify the relationship of the BTV and duodenum for each patient and its impact on dosimetric outcomes, an overlap volume (OLV) was created as the overlap between BTV and the PRV_duodenum.

**Fig. 1:**
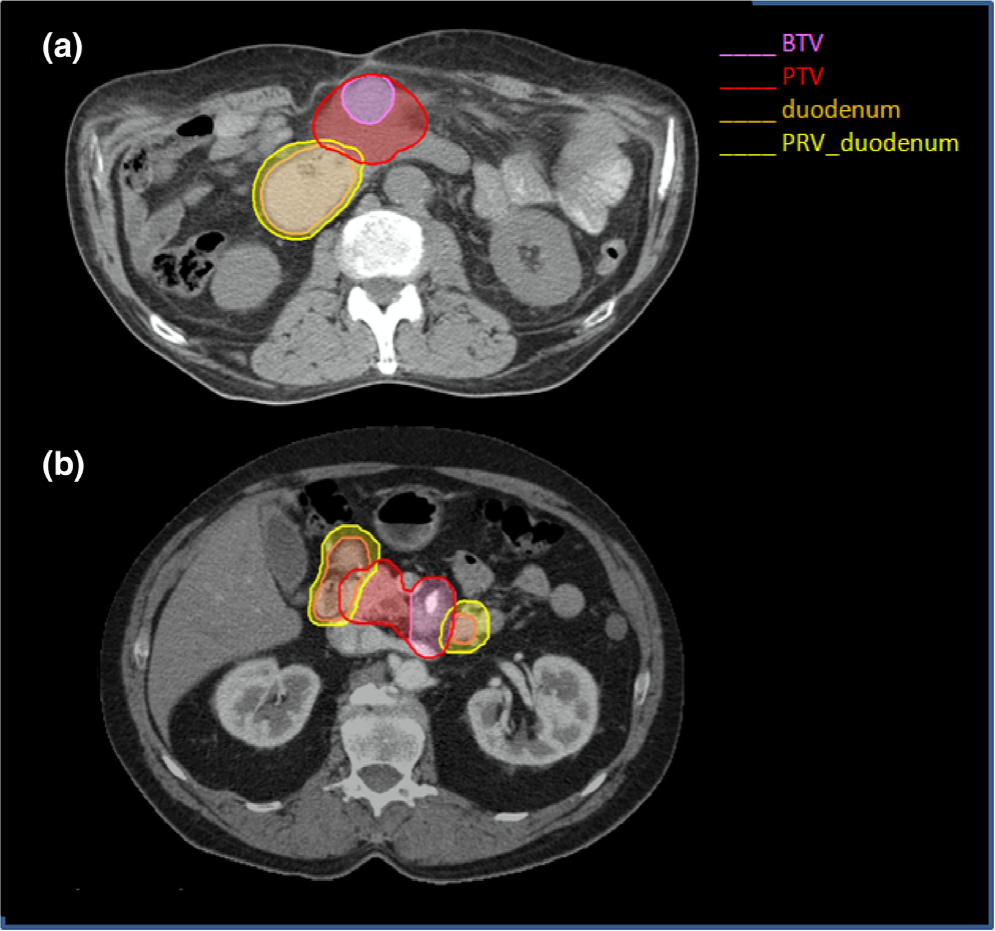
Axial slices of two patients showing the relationship among BTV, PTV, and PRV_duodenum.

Figure 1 shows the target, the OARs definition, and the OLV for a representative patient.

### 2.2 Dose prescription

For each patient three treatment plans were retrospectively calculated for each of conventional 6 MV FF and 6 MV FFF photon energies. At the first dose level, dose prescription was 30 Gy in 5 fractions (6 Gy/fraction) to PTV (including BTV). Then, two simultaneous different boost dose levels were prescribed to BTV: level2, 40 Gy (8 Gy/fraction) and level3, 50 Gy (10 Gy/fraction). In order to facilitate a comparison with treatments performed with a conventional fractionation (2 Gy/fraction), these doses were recalculated in terms of “Equivalent Dose in 2‐Gy fractions” (EQD2) using a α/β ratio equal to 10 for the tumor. The EQD2 to PTV and BTV were equal to 40.0 Gy at dose level 1; the equivalent doses to BTV at dose levels 2 and 3 were equal to 60.0 Gy and 83.3 Gy, respectively.

The primary goal for targets coverage was that 95% of BTV and PTV received 95% of their prescription doses. When this request cannot be fulfilled for BTV, a secondary goal demands that the BTV mean dose should be at last the 95% of prescription. Normal tissue constraints were based on the AAPM TG101 recommendations^24^ and are summarized as follows: PRV_duodenum and stomach: V_32Gy_ < 1 cc; Spinal cord: D_0.35cc_ < 23 Gy; liver: V_21Gy_ < 700 cc; kidneys: V_17.5Gy_ < 200 cc.

### 2.3 Treatment planning

Auto‐planning (AP) is a module in Pinnacle^3^ Version 16.0 designed to automate the inverse planning optimization process by utilizing a so‐called “Technique”, ie, a template of parameters that can be customized for each treatment protocol and tumor site.^25^

**Fig. 2:**
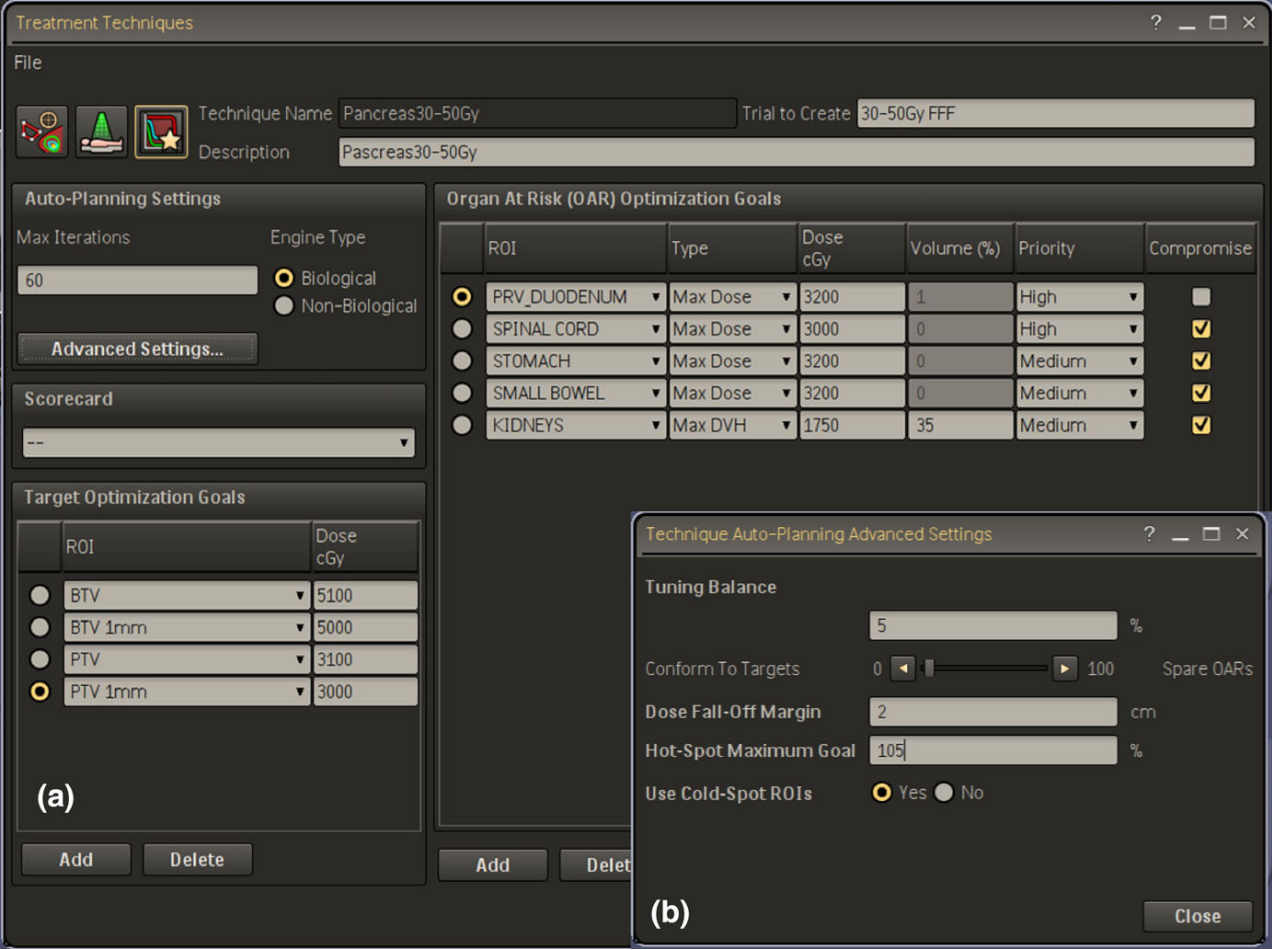
(a) Autoplanning setup template for pancreatic SBRT‐VMAT cases; (b) advanced settings template.

The Technique includes the definition of all beam parameters, dose prescriptions, and planning objectives for PTVs and OARs. The AP engine uses these definitions to iteratively optimize planning parameters to best meet the desired planning goals. During the optimization process, different kinds of dummy structures are automatically generated and new objectives to the planning goals are used in order to enhance the sparing of critical organs, the dose conformity, and the dose fall‐off outside the targets. The Technique adopted for the 12 patients with pancreatic carcinoma was defined as reported in the Figure 2, showing the values used for optimization. The objectives for the two PTVs were only defined by numbers close to prescription doses (in our experience we chose as target goals the prescription doses plus 1 Gy, so as to avoid possible under dosage in PTVs boundary). The OARs objectives included maximum dose, mean dose, and dose‐volume histogram points; they can have three different priority levels (high, medium, and low) and can be set compromised or uncompromised. The last choice was used for the PRV_duodenum, ie, when there is an overlap between PTVs and a serial OAR, and the OAR owns the overlapping voxels for the benefit of dose sparing. In the advanced settings template (figure 2b), we set up: (a) the tuning balance (ie, the balance between target dose conformity and OARs sparing), (b) the dose fall‐off margin (ie, the distance across which the dose should decrease from 80% to 20% in an automatically generated tuning ring structure around the PTVs), and (c) the Cold‐Spot ROI (i.e. the identification of cold regions inside the PTVs and the automatic creation of new tuning volumes and relative dose objectives to increase dose in the last optimization loops). Three patients, not included in the present series, were used to create and tweak the initial Techniques in order to generate plans fulfilling the clinical objectives.

For each patient, six plans were generated, one for each dose level, using the VMAT optimization process for both coplanar 6 MV_FF and 6 MV_FFF photon beams from an Elekta VersaHD linac (Elekta Ltd., Crawley, UK). Each plan consists of a dual arc; a gantry rotation is described by a sequence of 90 control points, ie, one every 4° around the patient with no overlap. All plans were calculated with the Pinnacle^3^ collapsed cone algorithm and a dose grid resolution of 2 mm.

### 2.4 Plans evaluation

Dosimetric quality of plans was evaluated by means of dose‐volume histograms (DVHs). According to the ICRU report 83,^26^ evaluated metrics for BTV and PTV were the minimal dose delivered to the 98% and 95% of the target volume (D98%, D95%), the median dose (D50%), and the maximum dose delivered to the 2% of the target volume (D2%).

Following the suggestion of Van’t Riet et al,^27^ we evaluated the dose conformity to both target volumes by means of the conformation number (CN) defined as:

$$\text{CN}=\frac{{\text{TV}}_{\text{RI}}}{\text{TV}}\times \frac{{\text{TV}}_{\text{RI}}}{V_{\text{RI}}}$$

where TV_RI_ is the target volume covered by the prescription isodose, V_RI_ is the volume of the prescription isodose and TV is the target volume. The reference isodoses are 95% of prescription doses for both BTV and PTV. CN values ranged from 0 to 1, where 1 was the ideal value. Larger CN values translate in smaller volume of the prescription dose delivered outside the PTV.

The integral dose (ID) was also calculated to evaluate the dose to normal tissues outside the PTV, considering uniform tissue density:

ID=mean dose×volume of normal tissue outside PTV

The ability of Autoplanning engine to create highly heterogeneous doses as requested in the present strategy (the highest dose to BTV while minimizing the doses to elective regions) was quantified by introducing a metric called dose contrast index.^28^ The ideal dose contrast (iDC) was defined as the ratio between the prescription doses to the BTV and the PTV. As the delivery of higher doses to the BTV increases doses to the surrounding PTV, we defined an actual dose contrast (aDC) as the mean dose to the BTV divided by the mean dose to the PTV (excluding BTV). The ratio of iDC and aDC defines the normalized dose contrast index (DCI) and quantifies the deviation of the actual aDC from the ideal iDC. A DCI value closer to 1 indicates a better dose contrast.

In addition, for all patients, the total number of monitor units (MUs) and the beam‐on‐time were recorded for both FF and FFF plans to assess treatment efficiency.

Differences between FF and FFF plans were quantified using the Wilcoxon matched‐pair signed rank with a statistical significance at *P* < 0.05.

### 2.5 Dosimetric verification

A detailed dosimetric verification of plans was performed in order to assess the deliverability of these complex treatments. Dose distributions were measured utilizing the 1500 2D ion‐chamber array together with the Octavius‐4D phantom both developed by PTW (PTW, Freiburg, Germany). The 1500 2D‐array consists of a matrix of 1405 ion chambers with a size of 4.4 mm × 4.4 mm × 3.0 mm. This array is inserted into the Octavius‐4D motorized cylindrical polystyrene phantom, capable to rotate synchronously with the gantry, so that the beam always hits the array in a perpendicular way, then allowing the possibility of three‐dimensional dose reconstruction and comparison. Measured and calculated dose distributions were compared by means of the gamma evaluation, based on the theoretical concept introduced by Low et al.^29^ Following the recent suggestions of the AAPM report No. 218,^30^ dosimetric verification was considered optimal if the percentage of points fulfilling gamma index criteria exceeded 95% using 3% for dose criterion and 2 mm for the distance‐to‐agreement criterion.

## 3 RESULTS

The average BTV and PTV volumes were 36.9 cm^3^ (range: 20.9‐58.1 cm^3^) and 109.5 cm^3^ (range: 41.6‐246.8 cm^3^), respectively. Tables 1, 2, 3 summarize the dosimetric results from DVHs analysis of BTV, PTV, and OARs. All plans were able to satisfy maximal OAR dose constraint for duodenum and other serial OARs at each dose level. Also dose‐volume constraints for parallel OARs were well within objectives for each dose level.

**Table 1:** Dosimetric comparisons for PTV and BTV coverage between FF and FFF plans.

	Dose level 1			Dose level 2			Dose level 3		
	FF	FFF	*P*	FF	FFF	*P*	FF	FFF	*P*
BTV									
V95% (%)	100.0 ± 0.0	100.0 ± 0.0	1.000	89.0 ± 11.3	89.5 ± 11.0	0.081	85.9 ± 12.0	86.0 ± 12.2	0.433
D98% (Gy)	30.8 ± 0.1	30.7 ± 0.1	0.272	34.7 ± 4.7	34.6 ± 4.6	0.790	37.5 ± 9.0	37.9 ± 9.1	0.136
D95% (Gy)	30.9 ± 0.1	30.8 ± 0.1	0.556	36.1 ± 4.3	36.1 ± 4.4	0.432	41.3 ± 8.5	41.4 ± 8.8	0.182
D2% (Gy)	31.6 ± 0.1	31.6 ± 0.2	0.175	42.6 ± 0.3	42.7 ± 0.2	0.146	53.6 ± 0.5	53.7 ± 0.6	0.209
Mean dose (Gy)	31.2 ± 0.1	31.2 ± 0.1	0.480	40.7 ± 1.1	40.8 ± 1.0	0.410	49.9 ± 1.9	49.9 ± 1.9	0.449
PTV									
V95% (%)	100.0 ± 0.0	100.0 ± 0.0	1.000	98.5 ± 3.4	98.5 ± 3.6	0.388	94.9 ± 6.5	94.9 ± 5.8	0.203
D98% (Gy)	30.7 ± 0.1	30.7 ± 0.1	0.694	29.3 ± 1.2	29.4 ± 1.2	0.555	27.9 ± 2.3	28.1 ± 1.7	0.347
D95% (Gy)	30.8 ± 0.1	30.8 ± 0.1	0.689	29.7 ± 1.0	29.8 ± 1.0	0.195	28.7 ± 1.6	29.0 ± 1.3	0.136
Mean dose (Gy)	31.2 ± 0.1	31.2 ± 0.1	0.529	35.4 ± 1.9	35.4 ± 1.9	0.555	39.4 ± 3.5	39.5 ± 3.5	0.351

**Table 2:** Dosimetric comparison for conformity and dose contrast indexes between FF and FFF plans.

	Dose level 1			Dose level 2			Dose level 3		
	FF	FFF	*P*	FF	FFF	*P*	FF	FFF	*P*
Conformation numbers									
CN BTV				0.592 ± 0.111	0.600 ± 0.094	0.084	0.664 ± 0.127	0.665 ± 0.119	0.885
CN PTV	0.677 ± 0.050	0.675 ± 0.049	0.136	0.564 ± 0.082	0.566 ± 0.083	0.875	0.455 ± 0.089	0.475 ± 0.105	0.069
Dose Contrast Indexes									
DCI				0.954 ± 0.037	0.955 ± 0.036	0.754	0.916 ± 0.050	0.916 ± 0.051	0.695

**Table 3:** Dosimetric comparisons for OARs coverage between FF and FFF plans.

	Dose level 1			Dose level 2			Dose level 3		
	FF	FFF	*P*	FF	FFF	*P*	FF	FFF	*P*
PRV_Duodenum									
V32Gy (cc)	0.0 ± 0.0	0.0 ± 0.0	1.000	0.5 ± 0.4	0.5 ± 0.4	0.314	0.7 ± 0.4	0.6 ± 0.4	0.130
Spinal Cord									
D0.35cc (Gy)	2.8 ± 1.5	2.7 ± 1.3	0.213	5.9 ± 2.0	6.0 ± 2.8	0.657	10.0 ± 5.2	10.1 ± 5.5	0.508
Stomach									
V32Gy (cc)	0.0 ± 0.0	0.0 ± 0.0	1.000	0.2 ± 0.4	0.2 ± 0.3	0.655	0.4 ± 0.5	0.3 ± 0.4	0.866
Liver									
V21Gy (cc)	4.4 ± 5.4	4.4 ± 5.4	0.917	7.1 ± 10.7	7.0 ± 9.3	0.345	11.7 ± 15.9	11.3 ± 16.7	0.600
Mean dose (Gy)	3.2 ± 1.1	2.9 ± 1.1	0.028	3.6 ± 1.2	3.1 ± 1.3	0.005	3.7 ± 1.4	3.4 ± 1.4	0.005
Kidneys									
V17.5Gy (cc)	0.0 ± 0.0	0.0 ± 0.0	0.180	2.0 ± 3.7	1.2 ± 3.1	0.817	7.9 ± 14.8	7.4 ± 14.4	0.327
Mean dose (Gy)	3.5 ± 0.9	3.2 ± 0.9	0.008	4.1 ± 1.2	3.7 ± 1.1	0.003	4.2 ± 1.4	3.8 ± 1.4	0.003
Healthy tissues									
I D (Gy*cc*10^5)	3.93 ± 0.97	3.81 ± 0.97	0.003	4.45 ± 1.01	4.23 ± 0.94	0.002	4.68 ± 1.06	4.45 ± 1.00	0.002

**Fig. 3:**
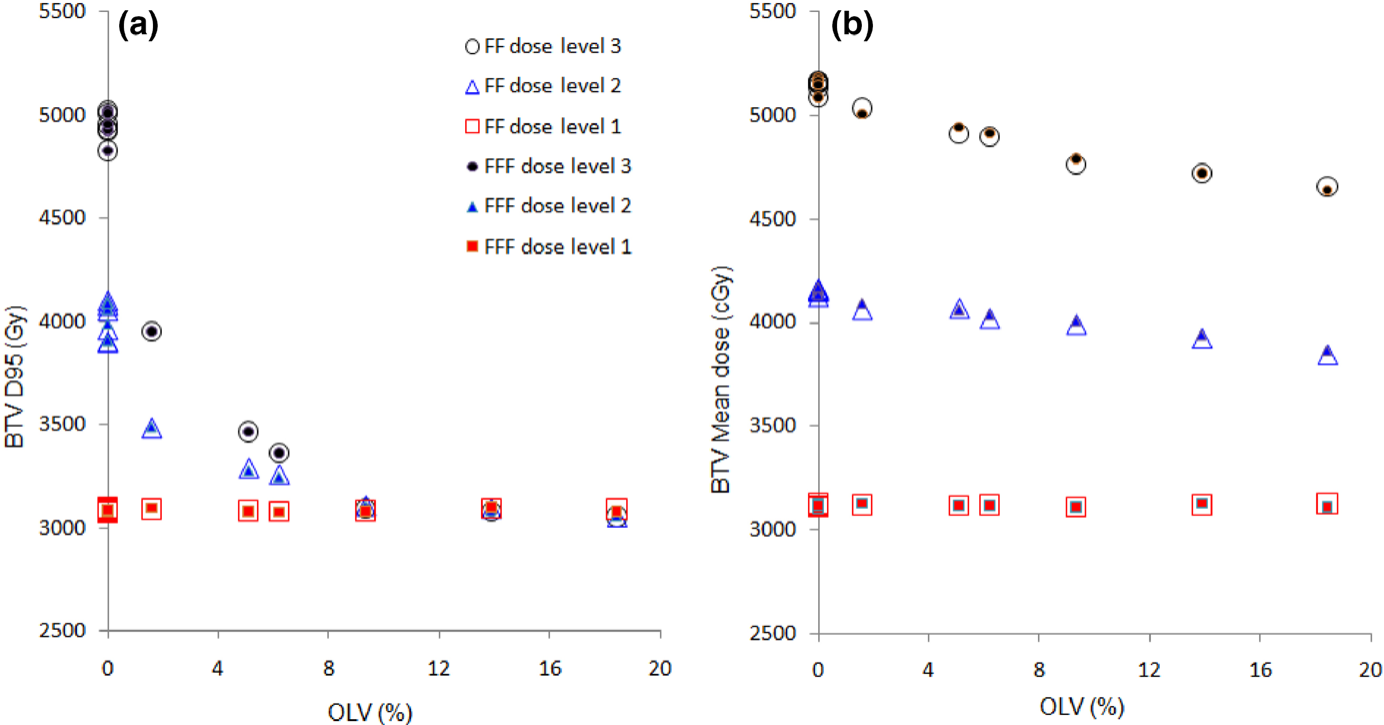
BTV D95% (a) and mean dose (b) as a function of overlap volume OLV.

All patients met dose objectives for PTV and BTV at dose level 1, both for FF and FFF beams. As we increased the BTV dose, less number of patients was able to meet BTV constraints in terms of D95%. The results showed that 6 (50%) patients met objectives for BTV D95 dose coverage at 40 and 50 Gy, respectively, satisfying maximal dose constraints for PRV_duodenum and other OARs. As expected, the analysis clearly shows that the BTV coverage (respecting all OARs constraints) decreased with increasing OLV, as reported in Figure 3a. In particular, when the OLV exceeded 8%, no advantage for BTV coverage in terms of D95% metric is observed despite the dose escalation. On the contrary, BTV mean dose was >90% of the prescribed dose for all patients, including those with major OLV (Figure 3b).

**Fig. 4:**
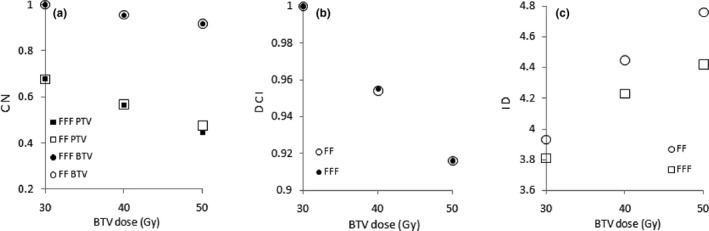
Conformation numbers (a), dose contrast indexes (b), and integral dose (c) of FF and FFF plans as a function of BTV dose.

The effect of the dose difference on the OARs was negligible between FF and FFF plans. As reported in Table 2, the CNs for PTV and BTV and the DCIs for the three dose levels of FF and FFF plans were similar, and differences were no statistically significant (p˃0.05 for all dose levels). Figures 4a and 4b show the CNs and DCI indexes of FF and FFF plans as a function of BTV dose; as expected, both CNs and DCIs decreased with increasing boost dose.

As reported in Table 3, the use of FFF plans resulted in a significantly reduced integral doses compared with FF plans, with *P* < 0.05 at all dose levels. In particular, FFF plans showed a ID reduction by 3.1% (*P* = 0.003), 4.9% (*P* = 0.002), and 4.9% (*P* = 0.002) for the three increasing dose levels. The Figure 4c shows the integral dose to normal tissues as a function of the BTV dose; the reduction in integral doses of FFF plans with respect to FF plans gradually becomes larger with increased BTV dose. In particular, when the BTV dose was escalated up to 50 Gy, the percentage increase in integral dose was 19.1% for FF plans and 16.8% for FFF plans.

**Fig. 5:**
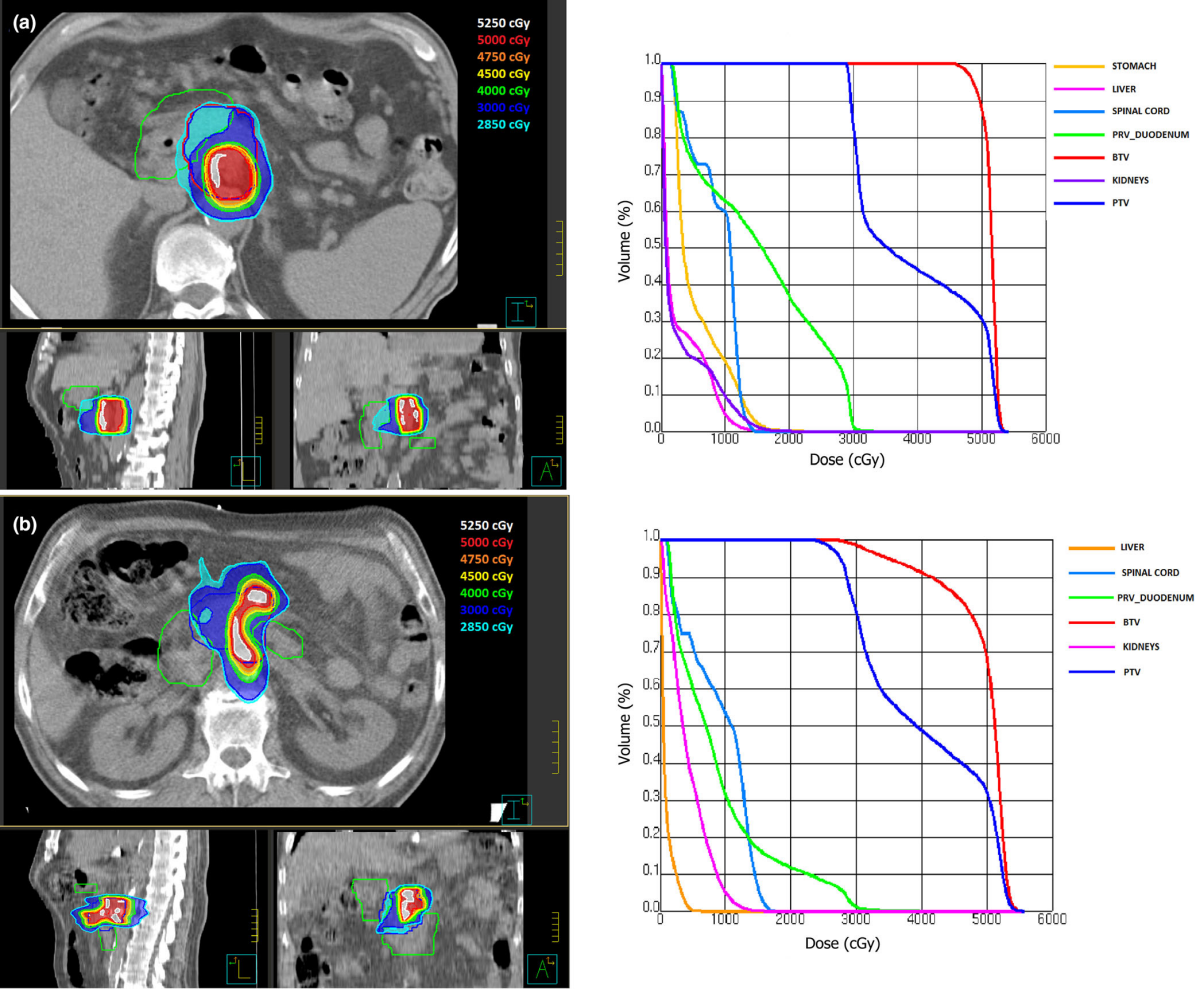
An example of isodose distributions and DVHs for two representative patients without (a) and with (b) overlap of BTV with PRV_duodentum, both planned at BTV dose of 50 Gy.

An example of dose distributions for two representative patients with and without OLV and planned at the highest dose level with FFF beams is shown in Figure 5.

Table 4 reports an overview of treatment delivery metrics for all plans. Compared to FF plans, a significant larger number of MUs was observed for FFF plans, reflecting an increased level of fluence modulation. For the three dose levels, the mean MUs ranged from 1191 to 2603 for the FF beams, and from 1320 to 2655 for the FFF beams, respectively. On the contrary, FFF beams exhibited significant reduced beam‐on‐times, ranging from 145 to 283 seconds for FF beams and from 131 to 139 seconds for the FFF beams, respectively.

**Table 4:** Summary of treatment efficiency in terms of MUs, beam‐on‐time, and pre‐treatment dose verification results.

	FF		FFF		*P*
	Mean	STD	Mean	STD	
Dose level 1					
MUs	1191	148	1320	163	0.003
Beam‐on‐time (sec)	145	6	131	4	0.003
γ pass‐rate (%)	99.6	0.6	99.2	0.9	0.225
Dose level 2					
MUs	1626	178	1750	164	0.003
Beam‐on‐time (sec)	208	8	137	4	0.003
γ pass‐rate (%)	99.0	0.6	98.5	1.0	0.128
Dose level 3					
MUs	2603	471	2655	445	0.374
Beam‐on‐time (sec)	283	11	139	4	0.003
γ pass‐rate (%)	98.5	0.9	97.5	1.2	0.089

Pre‐treatment verification was performed for all plans. With criteria equal to 3%‐2 mm for γ‐index analysis, the average γ% pass‐rate ranged between 98.5% and 99.6% for FF plans and 97.5% and 99.2% for FFF. γ% exceeded the acceptance threshold of 95% for all FF and FFF plans.

## 4 DISCUSSION

Radiotherapy of pancreatic adenocarcinoma represents a difficult therapeutic challenge. When treatment has a palliative intent, a moderate dose is usually prescribed to avoid radiation‐induced toxicity since the goal of therapy is to improve the quality of life. On the other side, when treatment has a neoadjuvant purpose, the main aim is to deliver high doses in order to obtain the tumor downstaging, especially in cases of vascular infiltration since vessels involvement is the main reason for unresectability. This study was, therefore, designed to answer a key question for pancreatic treatments: is a radiation treatment feasible and able to ensure to most patients the highest possibility to undergo radical resection together with a palliative effect with minimal toxicity risks? This goal can be pursued only if the planning optimization process is capable to produce strongly inhomogeneous dose distributions with very steep dose gradients at targets periphery. In particular, the integration of SBRT, VMAT, and SIB in a single therapeutic strategy represents an ideal scenario for this aim, but it constitutes a major challenge for planning optimization and led us to seek for further enhancement in the dose optimization algorithms by introducing automated planning in this difficult setting.

Recently, the Pinnacle^3^ Autoplanning engine has demonstrated several benefits in other complex anatomical sites as head‐neck cancers^18^, ^19^ and liver and lung SBRT,^21^, ^22^ improving the overall treatment planning quality and efficiency. In particular, in our clinic, we demonstrated that the implementation of Autoplanning for complex cancer cases planned with SIB strategy as head‐neck and high‐risk prostate cancers translated into a significant increase in dose conformity and reduction in integral dose.^31^ A recent paper addressed the feasibility of automated planning for pancreas SBRT using step‐and‐shoot IMRT technique.^32^ The authors reported that, also in this complex anatomical site, all plans met institutional dose constraints for OARs resulting in acceptable planning target volume coverage for all targets with different prescription levels. Based on the aforementioned studies, we applied the Pinnacle Autoplanning engine to the challenging planning optimization of SBRT treatments using SIB strategy for pancreatic cancer.

One of the main results of this study is that ablative doses up to 50 Gy in 5 fractions can be delivered to the region of vessel involvement to most patients, always keeping dose irradiation to OARs within specified limits. This result, as reported in Table 2, applies to both FF and FFF beams which show no significant differences for all dosimetric metrics for BTV and PTV coverage and OARs sparing.

As expected, the overlap of PRV_duodenum with the BTV was the most important predictor of BTV coverage, as reported in Figure 2. Our results showed that all patients with no overlap are candidable for this type of treatment at the highest dose level, with an optimal BTV coverage in terms of D95%. However, also patients with overlap received a much higher mean dose to BTV, with values greater than 46 Gy and 38 Gy for dose prescription of 50 Gy and 40 Gy to BTV, respectively. The patient with the greatest overlap (18.4%) reported the worst result, but in any case, the BTV mean dose of 46 Gy for dose level 3 demonstrated that strongly inhomogeneous dose distributions can be given also in very complex anatomical sites, where BTV partially overlaps PRV_duodenum. This result is of great interest because pancreatic tumors are particularly hypoxic at their core.^33^ Despite patients with partial overlap between PRV_duodenum and pancreas cannot reach full target coverage (ie, D95% ≥ 95% of PD), the possibility to irradiate high ablative doses to the hypoxic core may translate in major radiobiologic advantage. This concept is similar to the acceptance of dose heterogeneity within the PTV in other forms of treatments as recently proposed for prostate cancer.^34^

In this study we focused on the role of FFF beams on dosimetry and efficiency because of their potential favorable characteristics such as the higher dose rate and the lower peripheral dose coming from the absence of the flattening filter. FFF beams reported up to 11% higher monitor units compared to FF beams, due to the higher degree of modulation needed to manage the typical forward heterogeneous peak profile of FFF beams. However, the increase in MUs number did not translate in an increase in low‐dose volume irradiation; on the contrary, FFF beams showed a significant reduction in integral dose of 3‐5%. This outcome must be considered as an effect of the filter removal from the radiation path, leading to a reduction in leakage and scatter radiation.^35^

The major significant difference between FF and FFF beams was the beam‐on‐time. The average delivery time of FFF beam was about 2‐3 minutes, independently on fraction dose, with a reduction of 51% when compared with that of the FF beam at highest dose level. Since the use of FFF beams for pancreas SBRT enables the delivery of ablative doses in about only 2 minutes, potentially safer treatments due to the reduced intrafraction motion and patient movement error between setup and treatment completion are expected.

The plans obtained with Autoplanning in the present study are associated with a large number of monitor units and small and complex control points fields and shape. All these aspects contribute to a more complex fluence and may affect the dosimetric accuracy of actual delivery. Many studies reported that plan complexity indices are correlated with delivery accuracy and the quality assurance metrics.^36^ Then, with respect to the trade‐off between plan complexity and the dosimetric accuracy of the treatment delivery, we performed a “pre‐treatment” dose verification of all plans to assess the accuracy in dose delivery. Despite the complexity of these plans, the results of dosimetric verification confirmed the deliverability of our SBRT‐VMAT technique and its reliability for clinical applications.

A limitation of this feasibility study is that the effect of residual respiratory motion on dose distribution was not investigated. We are aware that the delivery of SBRT for pancreatic cases is complicated by tumor and normal tissue motion induced by respiration and that techniques for organ motion control are critical for successful implementation of this strategy. A recent study^37^ focused on the evaluation of motion mitigation techniques for pancreatic SBRT reported significant reductions in the average daily target motion. In particular, compared to no motion mitigation where displacement of pancreas can be as large as 20 mm, abdominal compression significantly reduced motion in anterior‐posterior and superior‐inferior directions to 5.3 mm and 8.5 mm, respectively. Another recent study^38^ focused on the quantification of allowable motion in dose escalation in pancreatic SBRT. The authors reported that mean allowable motions for 40 and 50 Gy dose escalation to the planning target volume should be less than 10.4 and 9.0 mm, respectively, in order to prevent significant deviations in target coverage and OARs sparing from the radiotherapy plan. The use of respiratory gating may be an even more effective strategy to reduce organ motion in pancreatic SBRT, and may allow a safer dose escalation through a reduction in target margin.

Finally, the choice of a dose of 30 Gy for PTV and higher doses up to 50 Gy in 5 fractions for BTV used in the present dose escalation feasibility study deserves some more detailed explanations. Using an α/β ratio of 3, a dose of 30 Gy to PTV corresponds to an EQD2 dose equal to 54 Gy for the late effects. This dose is still smaller with respect to the maximum tolerable dose for the duodenum (55 Gy). On the other hand, using an α/β ratio of 10 typical of acute responder tissues as tumors, it corresponds to an EQD2 dose equal to 40 Gy. This last dose can be considered sufficient to achieve a palliative effect in most patients. Morganti et al.^5^ demonstrated that a dose of 30 Gy in 10 fractions (EQD2: 32.5 Gy) translated in a complete response of pain in 50% of patients, with a partial response in a further 25% of patients. With regard to BTV, doses of 40 Gy and 50 Gy in 10 Gy fractions correspond to EQD2 doses of 60.0 Gy and 83.3 Gy for the tumor (using an α/β ratio of 10). These doses are higher compared to published studies^5^ for preoperative radiotherapy of pancreatic cancer and could be potentially more effective than standard regimens.

Given the ablative doses and the potential to injury, the translation in a clinical trial of the present strategy should be approached with a special attention to tumor and organ motion. Nowadays, MRI‐based image guidance has become a reality and the first experiences with MRI‐guided linear accelerators for the delivery of SBRT for pancreatic cancer have been recently reported.^39^ This new technology not only offers a superior soft tissue imaging compared to cone beam CT but also allows the opportunity for adaptive replanning when significant interfraction variation is highlighted,^40^ potentially increasing the safety and effectiveness of treatment.

## 5 CONCLUSIONS

In the present study, we evaluated the potential of automated planning to deliver ablative integrated boost doses to critical vasculature that limits resectability of pancreatic tumors. We reported that ablative doses up to 50 Gy in 5 fractions can be delivered to the BTV for almost all patients respecting all the normal tissue constraints. Autoplanning then can represent an effective way to generate complex treatment plans also in a SBRT strategy. Based on the promising aforementioned results, a prospective clinical trial for pancreas SBRT using automated planning with SIB‐VMAT technique and FFF beams seems to be justified.

## Conflict of interest

No conflict of interest.

## Authors’ contributions

Savino Cilla, Francesco Deodato, and Milly Buwenge conceived the study and mainly contributed in writing the manuscript; Anna Ianiro, Carmela Romano, and Gabriella Macchia performed planning optimization and data analyses; Pietro Viola contributed to dosimetric verification; Antonio Pierro and Silvia Cammelli conducted pancreas imaging and participate in data analyses; Vincenzo Valentini and Alessio G. Morganti provided overall guidance and revised the manuscript. All authors read and approved the final manuscript.
